# Sexual violence and suicide in Bangladesh

**DOI:** 10.1002/hsr2.269

**Published:** 2021-03-29

**Authors:** S. M. Yasir Arafat, Murad M. Khan

**Affiliations:** ^1^ Department of Psychiatry Enam Medical College and Hospital Dhaka Bangladesh; ^2^ Department of Psychiatry Aga Khan University Karachi Pakistan

Bangladesh, a South Asian country, is one of the highest densely populated countries in the world with about 160 million people. Suicide prevention has yet to get adequate attention in the country as it has no suicide surveillance system and national suicide prevention program. Suicide has been considered a criminal offense resulting in underreporting of suicides and scarcity of quality data.^1^, ^2^

The first case‐control psychological autopsy study in Bangladesh revealed mental disorders (OR = 15.33; CI = 4.76‐49.30), life events (OR = 17.75; CI = 6.48‐48.59), previous nonfatal attempts (OR = 65.28; CI = 0.75‐5644.48), and sexual harassment (OR = 12; CI = 1.56‐92.29) as the major risk factors for suicide in the country.^3^ Among the suicides, 61% were found to have at least one psychiatric illness (depression 44%) and 91% had life events where 40% were closely related to sexual and marital issues such as breaking engagement (9%), quarrel with spouse (8%), sexual harassment (8%), an extramarital love relationship (7%), an extramarital relationship of spouse (5%), child marriage and marriage against the will (2%), and quarrel with the lover (1%). Choice marriage was significantly found higher among suicides when compared to the age and sex‐matched living controls. The relatives of the deceased mentioned that 47% of the suicides were related to sexual and marital issues mentioned as extramarital love relationship (12%), premarital love relationship (12%), sexual abuse (9%), quarrel with spouse (6%), being refused to marry by the lover (6%), and forceful marriage (2%).^3^ Another previous case‐control study also identified a love relationship and a quarrel with the spouse as risk factors for suicide.^4^

The findings indicate a close relationship of psychosexual events with the suicides in Bangladesh, perhaps a sociocultural influence. Further studies are warranted to dig down the phenomenon and to find out a precise relationship. However, based on the status quo, prevention strategies targeting the cessation of sexual violence should be initiated, which needs multi‐sectoral attention. The government, nongovernment organizations, human rights activists, media, mental health experts, reproductive healthcare personnel, the forensic, and legal professionals should have an enduring liaison. A specialized coordinated crisis call center could help to release the distress and to find out the available resources needed for the sexual violence sufferers. Large‐scale awareness program should be started to stop sexual violence, child marriage, and disseminating the available way outs. Long‐term planned initiatives are warranted to identify and correct the maladaptive social construct regarding the love relationship, forceful marriage, child marriage, marriage after love relationship, and extramarital relationship. Certainly, selective strategies should be taken to improve the stress coping strategies for persons who are exposed to violence. The community mental health team and other gatekeepers can act as sentinel focus to train and monitor. Other worldwide tested suicide prevention strategies should also be tested in the country in a timely manner.^5^

## FUNDING

None.

## CONFLICT OF INTEREST

None.

## AUTHORS' CONTRIBUTIONS

Conceptualization: S.M. Yasir Arafat, Murad M. Khan.

Writing ‐ Original Draft Preparation: S.M. Yasir Arafat.

Writing ‐ Review & Editing: S.M. Yasir Arafat, Murad M. Khan.

S.M. Yasir Arafat and Murad M. Khan have read and approved the final version of the manuscript.

## Data Availability

Data sharing is not applicable to this article as no new data were created or analyzed in this study.
